# Barriers to the Use of Web-Based Mental Health Programs for Preventing Depression: Qualitative Study

**DOI:** 10.2196/16949

**Published:** 2021-07-15

**Authors:** Heidi Eccles, Molly Nannarone, Bonnie Lashewicz, Mark Attridge, Alain Marchand, Alice Aiken, Kendall Ho, JianLi Wang

**Affiliations:** 1 The Institute of Mental Health Research University of Ottawa Ottawa, ON Canada; 2 Department of Community Health Sciences Cumming School of Medicine University of Calgary Calgary, ON Canada; 3 Attridge Consulting, Inc Minneapolis, MN United States; 4 School of Industrial Relations University of Montreal Montreal, QC Canada; 5 Public Health Research Institute University of Montreal Montreal, QC Canada; 6 Faculty of Health Dalhousie University Halifax, NS Canada; 7 Department of Emergency Medicine University of British Columbia Vancouver, BC Canada; 8 Shandong Key Laboratory of Behavioral Medicine School of Mental Health Jining Medical University Jining China; 9 School of Epidemology and Public Health Faculty of Medicine University of Ottawa Ottawa, ON Canada

**Keywords:** prevention, mental health, depression, problem solving therapy, barriers, web-based program, qualitative study

## Abstract

**Background:**

Depression has a profound impact on population health. Although using web-based mental health programs to prevent depression has been found to be effective in decreasing depression incidence, there are obstacles preventing their use, as reflected by the low rates of use and adherence.

**Objective:**

The aims of the study are to understand the barriers to using web-based mental health programs for the prevention of depression and the possible dangers or concerns regarding the use of such programs.

**Methods:**

BroMatters and HardHat were two randomized controlled trials (RCTs) that evaluated the effectiveness of e–mental health programs for preventing workplace depression. In the BroMatters RCT, only working men who were at high risk of having a major depressive episode were included. The participants were assigned to either the control group or 1 of 2 intervention groups. The control participants had access to the general depression information on the BroMatters website. Intervention group 1 had access to BroMatters and BroHealth—the depression prevention program. Intervention group 2 had access to BroMatters and BroHealth along with weekly access to a qualified coach through telephone calls. The HardHat trial targeted both men and women at high risk of having a major depressive episode. The participants in the intervention group were given access to the HardHat depression prevention program (which included a web-based coach), whereas HardHat access was only granted to the control group once the study was completed. This qualitative study recruited male participants from the intervention groups of the two RCTs. A total of 2 groups of participants were recruited from the BroMatters study (after a baseline interview: n=41; 1 month after the RCT: n=20; 61/744, 8.2%), and 1 group was recruited from the HardHat RCT 1 month after the initial quantitative interview (9/103, 8.7%). Semistructured interviews were performed with the participants (70/847, 8.3%) and analyzed using content analysis.

**Results:**

There were both personal and program-level barriers to program use. The three personal barriers included time, stress level, and the perception of depression prevention. Content, functionality, and dangers were the program-level barriers to the use of web-based mental health programs. Large amounts of text and functionality issues within the programs decreased participants’ engagement. The dangers associated with web-based mental health programs included privacy breaches and inadequate help for severe symptoms.

**Conclusions:**

There are personal and program-level barriers to the use of web-based mental health programs. The stigmatization of help seeking for depression symptoms affects the time spent on the program, as does the public perception of depression. Certain barriers may be mitigated by program updates, whereas others may require a complete shift in the perception of depression prevention.

## Introduction

### Background

Major depressive episodes (MDEs) have a considerable impact on human health, with an annual prevalence of 4.7% in Canada [1], and they affect 350 million people worldwide [2]. MDEs cause workers to be less productive while at work (presenteeism) or completely absent from work (absenteeism). Presenteeism and absenteeism cost Canada approximately Can $2.5 billion (US $2.0 billion) and Can $6.8 billion (US $5.5 billion), respectively, in 2015 [3]. Preventive strategies have been shown to decrease the incidence of depression, thereby decreasing presenteeism and absenteeism [4,5].

Web-based mental health programs can play an important role in the prevention of depression by increasing accessibility, confidentiality, and sustainability [6], and those based on cognitive behavioral therapy (CBT) have been shown to be effective at decreasing stress [7]. A recent study found that web-based mental health programs were perceived by users to increase both individual and societal mental health awareness [8]. However, the rates of use and adherence to such programs can be low and inconsistent, with completion rates in randomized controlled trials (RCTs) ranging from 14% to 90% [9,10]. The frequency of completing web-based programs is lower than that in face-to-face therapies [10]. The level of use and adherence can have a direct impact on the effectiveness of e–mental health programs. Thus, it is important to understand the barriers associated with the use of e–mental health programs.

The demographic characteristics of age and sex seem to influence the use of web-based depression prevention resources. Web-based mental health programs are also less likely to be perceived as helpful by older populations [11]. Women and men favor different web-based mental health program designs and content [12], making it difficult to develop an inclusive program. In existing studies, the barriers associated with accessing web-based mental health programs include stigma, concerns regarding privacy, lack of time, impersonal nature, and a lack of perceived knowledge and confidence about web-based programs [11,13,14].

### Objectives

Although studies to date offer some insight into this topic, there are limited studies on the aversion to using web-based programs and examining other concerns or dangers that limit their use. The aims of this study are to (1) understand the barriers to using web-based mental health programs for the prevention of depression and (2) explore any possible dangers or concerns regarding the use of such programs.

## Methods

### Design

This qualitative study was embedded in two RCTs that each examined a different web-based program to prevent mental illness (BroHealth and HardHat, as described below). This project was approved by the research ethics board of the Royal Mental Health Centre in Ottawa, Canada.

#### BroHealth

BroHealth is a web-based program that was evaluated in the BroMatters RCT. It aims to reduce the risk of depression among working men at high risk of having an MDE. Details of the BroMatters RCT can be found in a previous publication [15]. The development of BroHealth was informed by the results of a national survey in the target population about the preferences of men classified as high risk for the design features of e–mental health programs [11]. The development was guided by a committee of research team members with expertise in psychiatry, epidemiology, eHealth, occupational psychology, addiction, information technology, and software programming. BroHealth includes modules on workplace depression, mental health information, and self-assessment tools. BroHealth is also linked to a UK-based CBT program, *Living Life to the Full*. Before finalization, BroHealth was pilot-tested among team members, stakeholder advisory committee members, and members of the general public recruited through personal networks and social media.

The participants in the BroMatters study were recruited using random digit dialing across Canada. These participants were working men at high risk of having an MDE. Their risk was calculated using a multivariable risk prediction (MVRP) algorithm, which is used to estimate the chances of developing depression in the next 4 years [16]. The participants were randomly assigned to 1 of 3 groups: group 1 had access to the BroHealth web-based program (intervention group 1), group 2 had access to both BroHealth and a coach (intervention group 2), and group 3 had no access to either the program or the coach but had access to general depression information on the BroMatters website (control group). The BroHealth program consists of self-checks, self-help modules, general depression information, and goal-setting materials. The participants in intervention group 2 could schedule optional telephone sessions with a coach once per week. Reminders were sent every other week through email to prompt program use in the intervention groups. The participants were interviewed at baseline, 6 months, and 12 months, and they were given a monetary incentive (Can $25 [US $20] gift card) to complete each interview.

#### HardHat

HardHat is an enhancement of BroHealth, targeting both men and women in the workplace. It encompasses enhanced visual design, depression information, self-assessment tools, and nine work-focused CBT and problem-solving therapy (PST) sessions designed by a psychiatrist with expertise in CBT and PST. Each session includes a 5-minute video recorded by professional voice actors in both English and French, in addition to in-class and/or homework assignments. Users are required to submit assignments for review and approval by a coach before moving on to the next session. Before finalization, HardHat was pilot-tested among 12 potential users from the community and revised based on feedback.

The target population of the HardHat RCT was working men and women who were at high risk of having an MDE but were not currently experiencing one. The risk of MDE was calculated using the same sex-specific MVRP algorithms [16] as the one used in the BroMatters RCT. The participants in the HardHat study were recruited through Green Shield Canada (GSC). A link was posted on the GSC website, and people were invited to complete an eligibility questionnaire. The eligible participants were randomly assigned to the intervention group (access to the HardHat program, including a coach) or a waitlisted control group that did not have access until after the final follow-up interview. The HardHat program includes five compulsory sessions and four supplementary sessions. The sessions are based on the principles of CBT and PST and are designed to help participants work through the problems that cause them stress. Each participant was assigned a coach who was available through the chat function to assist them throughout the program and answer any questions. To encourage the use of the program, emails were sent every other week as a reminder. As in the BroMatters study, each participant completed three structured interviews and was given a Can $25 (US $20) gift card at the end of each interview. The HardHat participants were also given incentives to complete the program’s sessions, unlike those who participated in the BroMatters study. To complete each session, the HardHat participants were given 100 GSC points and were included in a monthly draw to win a Can $100 (US $80) Amazon gift card.

### Participant Recruitment for Qualitative Study

Originally, the qualitative interviews were only planned to be conducted after the completion of the BroMatters RCT; however, because of low use throughout the duration of both RCTs, an additional round of interviews was conducted to investigate the reasons for the low use. The participants for this qualitative study were randomly selected from the intervention groups (ie, those given access to BroHealth or HardHat). A total of 3 different groups were recruited from the 3 different sample sets. Group 1 consisted of participants from the BroMatters RCT who had little or no use of BroHealth (maximum of one log-in). The interviews were conducted 1 month after the RCT began. Participants were randomly selected and interviewed until 41 participants were reached and code saturation was reached within these interviews. Group 2 was recruited after the BroMatters RCT was completed. Overall, 20 of these participants were randomly selected from either of the intervention groups and interviewed, and code saturation was reached within these interviews. The total population size for groups 1 and 2 was 744 individuals; thus, our use of 61 participants for qualitative data collection represents 8.2% (61/744) of the total population of participants in the BroMatters source study. Group 3 was recruited from the HardHat RCT 1 month after the study began; this study had a total of 103 participants to sample from. The participants were randomly selected if they had a maximum of one log-in, and they were interviewed until code saturation was reached. To maintain the homogeneity of the study sample for this analysis, we included nine interviews conducted with male participants from the HardHat trial, representing 8.7% (9/103) of the HardHat population.

### Data Collection

The semistructured interview guides were designed by the team members; different interview guides were used for each group, but the questions included similar topics. The questions focused on the lack of program use, motivations, and program perception. The qualitative interview guides are included in Multimedia Appendix 1. The telephone interviews were conducted in English or French by research staff not involved in the RCT data collection. The average length of the interviews was 9 minutes. All interviews were audio recorded and transcribed verbatim by the members of the research team.

### Sample Size Justification

The principle of data saturation was used to determine the sample size for this qualitative study. Data saturation allows for a complete picture to be formed about participant perceptions on the topic. For this study, data saturation was defined as *the point where no novel information relevant to the topic is being gathered in the interviews*. To ensure that the sample would reach data saturation, the study by Sim et al [17] was drawn upon to determine appropriate sample sizes. They inferred that many qualitative research projects reach data saturation with between 10 and 40 participants. A rough sample size of 41 was chosen for the first set of qualitative interviews (group 1: BroMatters low use); once the 41 interviews were completed, it was realized that saturation occurred well before the final interview. Therefore, a sample size of 20 participants was chosen for groups 2 and 3; in both cases, saturation was reached within these interviews.

### Data Analysis

Inductive content analysis was conducted using NVivo version 12 (QSR International) [18,19]. Content analysis has been effectively used in various mental health research studies to understand perceptions of symptoms and treatment interventions [20]. The second author (MN) read several of the transcripts and identified key findings that were of interest, after which the first author (HE) read all transcripts multiple times to become familiar with the data. Subsequently, HE performed open coding to identify themes in the data, and short phrases (ie, codes) were developed to represent each theme. Next, HE amalgamated the codes into broader categories to develop a coding framework. The codes did not need to be reported in a set number of interviews to be included in the framework because of the semistructured nature of the interviews; interviews that follow a semistructured design may vary from participant to participant, and unique questions may arise that may not have been part of the broad interview guide. Next, a third researcher assessed the coding framework to ensure that it fully represented the interview themes. At this point, the authors noted that the categories fell into two very general groups and were therefore categorized one more time. Subsequently, the transcripts were recoded by HE using the final framework. Finally, the coding framework prepared by HE was compared with a preliminary analysis performed by MN for consistencies.

## Results

### Overview

A total of 70 participants completed the semistructured telephone interviews—61 and 9 participants from the BroMatters and HardHat RCTs, respectively. The demographic characteristics of each group are presented in Table 1. Although the population sizes for each program were very different, the demographics were very similar. The average ages of the participants in the HardHat, BroMatters low use, and BroMatters post-RCT studies were 43.0, 39.5, and 42.7 years, respectively, and most participants in all these studies resided in Ontario. The average program use, measured in terms of log-ins, of all participants in the BroMatters study was 1.75 log-ins; similarly, most HardHat participants did not complete the program. Furthermore, the average depression risk scores calculated by using the MVRP algorithms were 32.3 and 19.8 in HardHat and BroMatters, respectively.

A total of 6 barriers to using web-based mental health programs were described. These six barriers were categorized as personal-level barriers that revolved around the personality of the user and program-level barriers that were specific to the web-based program itself. The personal-level barriers included lack of time, level of stress, and disbelief in prevention, whereas the program-level barriers included content complexity and redundancy, program functionality, and perceived dangers (Textbox 1).

**Table 1:** Demographics for the participants in the different participant groups (N=70).

Demographics			All participants (N=70)		BroMatters low use (n=41)		BroMatters after RCT^a^ (n=20)		HardHat, (n=9)
Age (years), mean (range)			40.6 (20-67)		39.5 (20-63)		42.7 (27-66)		43.0 (22-67)
**Location, n (%)**									
	British Columbia	12 (17)		7 (17)		3 (15)		2 (22)	
	Alberta	8 (11)		5 (12)		3 (15)		0 (0)	
	Saskatchewan	4 (6)		3 (7)		1 (5)		0 (0)	
	Manitoba	1 (1)		1 (2)		0 (0)		0 (0)	
	Ontario	35 (50)		21 (51)		7 (35)		7 (78)	
	Quebec	5 (7)		1 (2)		4 (20)		0 (0)	
	New Brunswick	1 (1)		0 (0)		1 (5)		0 (0)	
	Nova Scotia	4 (6)		3 (7)		1 (5)		0 (0)	
Depression risk score (%), mean (range)			23.1 (7-84)		19.2 (7-84)		20.4 (7-84)		32.3 (22-67)
Number of log-ins, mean (range)			—^b^		1.2 (0-11)		2.3 (1-8)		—
**Completer, n (%)**									
	Complete	—		—		—		2 (22)	
	Partially	—		—		—		3 (33)	
	Not started	—		—		—		4 (44)	

^a^RCT: randomized controlled trial.

^b^Not available.

**Summarization of resulting categories.**

**Personal barriers**

- Lack of time
  - Life is busy.
  - People do not prioritize stress prevention.
- Level of stress
  - Too little stress, and people do not think they need it
  - Too much stress, and people cannot find the time and motivation
- Disbelief in prevention
  - Stigma
  - People do not focus on depression.

**Program barriers**

- Content complexity and redundancy
  - Text heavy
  - People do not like activities in program
  - Redundant information
- Program functionality
  - Disorganized flow
  - Broken links
  - Internet as a medium
- Perceived dangers
  - Privacy
  - Using it as a treatment instead of seeking help

### Personal Barrier

#### Lack of Time

Most participants reported a lack of time or an inability to prioritize use as the largest barrier to program use. For instance, one participant said the following:

...it’s in my list of things to get to but life is like super busy, I have a young kid and a newish job and so it’s there, but I haven’t gotten to it.

BroMatters low use; age: 42 years

Prioritizing other aspects of their lives often caused the participants to forget about the program entirely. A participant commented as follows:

...I keep forgetting. It’s in the pile of things to do and I keep forgetting to go back and look at it again.

BroMatters low use; age: 51 years

#### Level of Stress

The level of participant stress, both high and low, emerged as a barrier to program use. Some participants neglected to use the web-based programs because they believed that they did not need to use it:

I didn’t really need anything because work’s going pretty well.

BroMatters low use; age: 32 years

Others managed their stress well using other support, including therapy, medication, and self-management strategies. For instance, one participant mentioned not using the program:

...because I currently have a psychologist...and I’ve been meeting with that doctor on a regular basis.

BroMatters low use; age: 36 years

The participants may also have been disinclined to use the program because they were struggling to manage their mental health; hence, they felt overwhelmed and unable to avail themselves of the web-based program. For instance, one participant said the following:

...over the past month...my mental state of mind has deteriorated. I just got no determination or willpower to do anything.

HardHat; age: 55 years

These participants were often overwhelmed by the content when they tried to use the programs. One participant explained as follows:

I was already pretty stressed out and a little overwhelmed and had high levels of anxiety as a result, and so when I got to the website and saw the information that there was, I had to like pull back because it was too much.

BroMatters post-RCT; age: 42 years

#### Disbelief in Prevention

In multiple cases, participants were skeptical as to whether anyone would use the program as a preventive measure before developing symptoms of depression:

I don’t think people are going to use the resource before they have depression...people don’t turn to something until they have it.

BroMatters low use; age: 36 years

Program use as a preventive measure was also hindered by the stigma associated with getting mental health help. One participant stated:

There’s some stigma attached to the whole idea of not being mentally well, so I think it’s something that people like to keep private.

BroMatters low use; age: 43 years

### Program Barrier

#### Content Complexity or Redundancy

Content that is difficult to digest or redundant when considering the information available elsewhere is a barrier to program use. Some participants felt that the programs were too text heavy, thus deterring participation and causing them to lose interest in the program. One participant remarked the following:

I think what it comes down to is the amount of reading. If there is a lot of reading, it’s just kind of a put off.

BroMatters post-RCT; age: 47 years

Some participants felt that the modules were too long to complete in one sitting, and they neglected to either start the modules or return to complete them. In addition, some participants found the program content to be redundant when considering other resources that they had access to elsewhere, leading to the limited use of the programs. One participant explained this as follows:

...after a half a dozen times I found that the content was redundant with what I had already gotten from my healthcare provider.

BroMatters post-RCT; age: 37 years

For some participants, the types of activities did not align well with their preferences. For example, one participant said the following:

I’m not the type of person to divide a problem up into six component pieces.... My personality is a type where I don’t care about how many different parts of the problem there are.

HardHat; age: 67 years

#### Functionality

Another topic discussed by the participants was how the program functioned. Functionality issues such as broken links and disorganized flow discouraged the use of the programs. When the participants had difficulty using the program, they felt discouraged and were less likely to return to it. The internet itself was also perceived to be problematic as a medium for the delivery of mental health prevention resources. Some participants did not like using the internet at all or did not use it often enough. Many participants also had an aversion to using the internet as a resource for mental health prevention. For instance, one participant said the following: “When you’re stressed out or something like that, last thing you want to do is go on your phone and tell your phone how stressed you are.” [BroMatters post-RCT; age: 47 years].

#### Perceived Dangers

Overall, very few participants reported concerns about personal dangers or negative impacts of web-based mental health programs. A few of the participants voiced concerns about privacy. For example, one participant described how navigating contemporary times that are pervaded by telephone and email scams leaves them skeptical about the legitimacy of certain websites. For instance, a participant commented as follows: “...there’s so many traps for an old man to fall into” (BroMatters low use; age: 63 years). Furthermore, he provided additional context: “...99% of these emails and phone calls and people at my door are scammers wanting money” (BroMatters low use; age: 63 years). In addition, the programs’ focus on personal mental health left some participants concerned about privacy:

I’m a very private person and so the fact that I opened up as much as I did in the first call kind of concerned me, and so I’m hesitant just to spill my guts when I don’t really know who it is I’m talking to.

BroMatters low use; age: 48 years

The other danger that the participants conveyed was the possibility that individuals with severe symptoms may use the program at the expense of specialized treatment and thus may not receive the care that they need. One participant explained this as follows:

...if somebody would try to use this to replace seeking other help or talking to someone else about their issues.

BroMatters post-RCT; age: 46 years

### Group Differences

Most categories were consistent across the interview groups and programs. However, the participants who were interviewed only 1 month after the RCT started (both BroMatters and HardHat) reported *time* as the biggest barrier to program use, whereas the participants who had completed more of the program and who were interviewed after the RCT (BroMatters only) was completed stated that *need* was the biggest barrier. Moreover, the BroMatters participants had greater difficulty with program functionality and flow than the HardHat participants, which was in part due to the fact that the CBT program (*Living Life to the Full*) in the BroHealth program was externally linked. The challenges identified by the HardHat participants focused more on not receiving emails and not understanding how to access the site.

## Discussion

### Principal Findings

This study found that barriers to program use exist at both personal and program levels. Personal-level barriers affected initial use of the program. Many participants did not prioritize their mental health, citing that time constraints or a busy lifestyle did not allow them the time to use the program. This led many participants to believe that depression cannot be prevented. Although all participants in the study had a high risk of MDE, some did not believe that they had a stressful life and therefore did not believe that they needed to use the program. The barrier of continued use was discussed when the program itself was up for discussion. Content had a large effect on continued use. When content was perceived as redundant or not applicable to the participants, they discontinued use. Similarly, if program use caused frustration because of technical problems or the participants’ dislike of the internet as a medium, they were less likely to use the programs. Some participants were also concerned about the possibility of privacy breaches resulting from program use.

The results of our study are consistent with previous research in terms of personal and program-level barriers (lack of time, stress, content and medium, and privacy concerns) [21-23]. One important difference between our study and previous research is that BroHealth and HardHat were designed for prevention purposes (rather than treatment), and there are limited studies on the lack of interest in preventing depression as a barrier to program use. This is an important finding that highlights a significant gap in the current knowledge of web-based mental health programs for preventing depression.

The design and development of BroHealth and HardHat were informed by a large national survey and several rounds of usability testing. Nevertheless, the use of these programs was not optimal. This qualitative study uncovered several barriers to web-based program use and has significant implications for mental health. Lack of time and perceived stress are the main personal barriers. It should be noted that the RCTs evaluating these two web-based programs were conducted from 2017 to 2019. The reported barriers reflect how potential users perceived web-based mental health programs during that time. Such barriers may be different in the context of COVID-19 with the closure of in-person health services [24]; implementation of web-based health services [25-27]; physical distancing; isolation; and widely reported feelings of loneliness, depression, and anxiety [28] due to these public health measures. The COVID-19 crisis and public health restrictions could accelerate the use of mobile and digital health [29]. Therefore, studies on this topic, both during the pandemic and in the postpandemic era, are needed. BroHealth functionality and technological problems were related to an external link to a CBT program. Such a linkage should be avoided in future web-based programs or apps because technical issues may occur in unpredictable ways. Other program functionality issues were also evident, highlighting the importance of a user-friendly design and interface. In addition, many HardHat participants did not receive the reminder emails that were sent, thus limiting their effectiveness [8]. The qualitative results also shed light on the importance of program personalization and balancing text and video and audio content, and these findings should be taken into consideration in the design of future digital mental health programs. Adapting web-based mental health programs into web-based or mobile app personal informatics (PI) systems has the potential to mitigate some barriers to program use. PI systems, which collect and store personal data that can be accessed by users for a number of purposes, would improve the need for program personalization. PI systems can be websites and mobile apps that can be seamlessly integrated into daily life, thus improving engagement. One salient example of the positive effects of PI systems is activity trackers that users can use to monitor their activity level, set goals, and monitor progress; such trackers have been shown to improve physical activity among users [30].

Many participants believed that depression cannot be prevented. This mistaken view leads to a reluctance to use programs aimed at prevention, including web-based mental health programs. This finding is echoed in previous studies [11,31-33]. People who believe that depression cannot be prevented tend to prioritize other activities over their mental health, perhaps because of the lack of emphasis that society places on mental health and social norms related to the male sex. To mitigate barriers to program use, program modifications alone are not sufficient; a shift toward depression being perceived as a preventable illness is imperative, especially among men. Important steps would be for society to prioritize mental health alongside physical health, raise awareness about mental health, increase mental health literacy, and develop effective depression-risk communication. Such population-level mental health education and promotion efforts may improve societal acceptance of the notion of prevention so that maintaining good mental health would become a more mainstream and socially acceptable priority. For example, the historic change in how breast cancer prevention has progressed from a private issue to a widely promoted public health goal could be considered a relevant precedent. This study contributes to the first step by identifying depression as a continuing problem in the mental health field. Regardless of the effort put into identifying motivators and effective programs, for people to choose to partake in such programs, mental health must be made a top priority in society. Increased focus on mental health at all levels of government and at workplaces is a good first step to increase the perceived importance of mental health. An increase in government resources allocated to digital mental health care and preventive services on the web in conjunction with the application of artificial intelligence technologies could improve the promotion of mental health and better adaptation. As people spend many hours at work every day, workplaces are an ideal environment to promote positive mental health and implement digital preventive mental health programs.

### Limitations

This study includes some limitations. First, some participants were interviewed a number of months after having used the program. As such, for some, recalling the program details was difficult. In addition, because demographic data were not collected, the results of this study cannot be extrapolated to larger populations. Furthermore, the barriers described in this study were specific to the BroHealth and HardHat programs; therefore, the barriers in this study, such as functionality, reminders, and content, cannot be generalized to other programs.

### Conclusions

Although the use of web-based mental health programs to decrease the prevalence and incidence of depression has been shown quantitatively to be effective, there are barriers to their use. Barriers such as time and the perception of depression prevention need to be changed at the population level. Increasing the perceived importance and priority of depression prevention is likely to mitigate these barriers; therefore, research into these areas is imperative. Having easy-to-use programs with minimal text may improve engagement with web-based mental health programs, especially among those who may have had difficulty with previous attempts to use such programs.

## Appendix

Multimedia Appendix 1 Qualitative interview guides.

## Abbreviations

CBT: cognitive behavioral therapy
GSC: Green Shield Canada
MDE: major depressive episode
MVRP: multivariable risk prediction
PI: personal informatics
PST: problem-solving therapy
RCT: randomized controlled trial
