# Supplementary material for: Barriers to the Use of Web-Based Mental Health Programs for Preventing Depression: Qualitative Study
Source: JMIR Form Res. 2021 Jul 15;5(7):e16949. doi: 10.2196/16949 (PMC8323021; doi:10.2196/16949)
Supplement: Multimedia Appendix 1 [file formative_v5i7e16949_app1.docx]

Qualitative Interview Guides

This is a Multimedia Appendix to a full manuscript published in the J Med Internet Res. For full copyright and citation information see http://dx.doi.org/10.2196/jmir.16961

BroHealth Low use

1. Did you know you had access to an e-mental health service called BroHealth? ( if they are in group 3 I will add coaching as well)
2. Have you ever used BroHealth to help manage your stress at work?

- Used once
  - Why have not you used it again?
    - You don’t have time? Please elaborate
    - You are not interested in it?
    - You are concerned others would know?
    - You don’t need such help?
    - The program is difficult to use
    - The features are not attractive
- Have not used it (go to question #3)

1. Why do you think you haven’t used it as much?

- Was it due to lack of time?, please elaborate
- Was it due to lack of interest? please elaborate
- You are concerned others would know?
- You don’t need such help?

1. In your opinion, how can we motivate people like yourself to use these services?
2. Do you think e-mental services are an effective strategy to prevent depression at the workplace? Why or why not?

BroHealth After RCT

[**Note:** Intervention Group 2 has access to BroHealth; Intervention Group 3 has access to BroHealth and work-life coaching]

1. When you first enrolled in the study, we sent you an email with your login information for the BroHealth website. Do you remember getting this email? [If no, skip to Question 8]
2. Did you ever log in to BroHealth? If yes, how many times? [if no, skip to Question 8]
   1. If only used once – why?
      1. Did you complete any modules?
      2. Did you spend at least 10 minutes interacting with it?
      3. Why do you think you never logged on again after that?
   2. If used more than once – what made you come back?
3. When using BroHealth, what was your experience like?
   1. What were your impressions of the website?
   2. Do you remember what parts of the website you looked at?
      1. Did anything stand out to you?
   3. Overall did you find the website easy to use?
      1. If yes, what in particular did you find easy to use?
      2. Were any parts confusing or unhelpful? If so, please describe these.
   4. Do you have feedback about the content? [Too much? Too little? Appropriate? Engaging? Suggestions for including additional content?]
4. Overall, was BroHealth helpful to you?
   1. Yes – how? What parts were helpful?
   2. Were any parts unhelpful?
5. Do you think that there are any possible dangers or disadvantages of using BroHealth?
   1. Did BroHealth have any negative impacts on you personally?

**[GROUP 2 SKIP TO QUESTION 8]**

1. **[group 3 only]** Did you know that you had access to a free telephone coaching service? [if no, skip to d.]
   1. Did you use this service?
   2. If yes – what was your experience like? Was the coaching helpful? [probe for details re: usefulness, ease of use, comfort with coach, knowledge of coach, etc.]
   3. If no, what kept you from using this service?
   4. Have you ever used another web or mobile-based coaching program?
      1. Can you tell me a bit about the program you used?
      2. Was that program easy to use? How so?
      3. Was that program helpful? Why?
      4. What attracted you to that program? What kept you coming back?
      5. What features did you like most? Did you dislike any features?
2. Have you ever used any other online or web based program similar to BroHealth? [if no, skip to Question 8]
   - 1. Can you tell me a bit about the program that you used?
     2. Was that program easy to use? How so?
     3. Was it helpful? Why?
     4. What attracted you to that program? What kept you coming back?
     5. What features did you like most? Did you dislike any features?
3. What do you think of when you hear the term “mental health”?
   1. Do you think there is a better term that might be more clear? More comfortable for people to discuss? Less stigmatizing?
4. Is there anything else that you’d like to add that we haven’t already talked about?

HardHat RCT

1. Did you have a chance to log in to the HardHat website?
   1. Never logged in
      1. Why do you think you never log onto the program? (skip to Q5)
   2. Logged in once
      1. Why do you think you never logged on again after that?
2. When using the HH program, what was your experience like?
   1. What were your impressions of the program?
   2. Was the content appropriate for you? why/why not? (ask them to elaborate)
      1. Do you have any ideas where the content would be more appropriate?
      2. did you find the program helpful? Why/ why not? Why/why not?
   3. Overall did you find the program easy to use?
      1. If yes, what in particular did you find easy to use?
      2. Were any parts confusing or unhelpful? If so, please describe these.
   4. Do you have any feedback about the program? (i.e. engaging, appropriate content, too much / too little information)
3. Do you think that the program was unhelpful in any way or are there disadvantages of using HardHat?
   1. Did HardHat have any negative impacts on you personally?
4. Did you know that you had access to an in-program coach?
   1. Did you interact with the coach at all?
      1. If yes, what was your experience like?
      2. Was the coaching helpful (probe for details re: usefulness, ease of use, comfort with coach, knowledge of coach, etc.)
   2. If no, what kept you from using this service?
5. Do you remember getting weekly reminder emails?
   1. Yes – did that motivate you to use the program?
      1. Why/why not?
   2. No – confirm email address
6. What do you think would motivate you to use the program (or to use the program more)?
   1. Do you think that text message reminders would be helpful? Why/why not?
   2. Do you think that video reminders sent by email would be helpful? Why/why not?
   3. If your coach contacting you prior to you starting the program would that be helpful? Why/Why not?
   4. Did you know that you receive 100 GSC points after completing each HardHat session and you also have a chance to win a 100$ Amazon Gift card at the end of every month?
      1. Yes- did this motivate you to use the program?
      2. No (did not know)- will this motivate you to use the program?
7. Now I’m going to tell you a bit about something called motivational interviewing. Motivational interviewing helps you to think differently about a behavior and to consider the advantages of changing that behaviour. This will be conducted through the HardHat chat function with a coach informing you on reasons why it would be useful for you to use the program etc. Do you think that motivational interviewing would help motivate people to engage more with the HardHat program?
   1. Why/why not
8. Is there anything else that you would like to add that we haven’t already talked about?
